# The Impact of the COVID-19 Pandemic on Diagnosing and Treating Attention Deficit Hyperactivity Disorder: New Challenges on Initializing and Optimizing Pharmacological Treatment

**DOI:** 10.3389/fpsyt.2022.852664

**Published:** 2022-04-06

**Authors:** Daniel Segenreich

**Affiliations:** Petrópolis Medical School, Rio de Janeiro, Brazil

**Keywords:** attention deficit-hyperactivity disorder, treatment, COVID-19, ADHD, pharmacological treatment

## Abstract

**Introduction:**

COVID-19 has been causing huge disruptions in mental healthcare services worldwide, including those related to ADHD. Some consequences of the pandemic, such as virtual schooling and remote work, as well as increased telemedicine, have posed new challenges for ADHD diagnosis and treatment. In this narrative review, we summarize existing COVID-19 and ADHD literature especially focusing on ADHD diagnostic during the pandemic and treatment adherence.

**Methods:**

The databases searched were: PubMed, PsycINFO, EMBASE, Google Scholar and medRxiv. We included all English language articles and preprints that reported on medication/pharmacological treatment among the terms “ADHD” and “COVID-19” resulting in a total of 546 articles. The final search was done on Dec-23 2021. We selected fifteen articles focusing on the challenges of ADHD diagnostic during COVID-19 pandemic.

**Results:**

Of the fifteen studies included, most were cross-sectional and perspective pieces. Most of them discussed that individuals with ADHD present risk factors that may make them more vulnerable to health negative consequences of the pandemic, which in turn may have an impact on treatment efficacy and adherence. Telemedicine is also addressed as a potential powerful instrument on monitoring ADHD treatment.

**Conclusion:**

Despite the challenges posed by the pandemic on monitoring ADHD treatment, the available literature stressed that the current scenario also may offer new opportunities that could lead to the development of individualized treatment interventions, such as the remote monitoring of symptoms.

## Introduction

Attention deficit hyperactivity disorder (ADHD) is a prevalent, impairing condition that is frequently comorbid with other psychiatric disorders and creates a substantial burden for the individual, their family, and the community (1). Positive correlations between ADHD diagnosis and unsafe school, unsafe neighborhood, and economic hardship are evident in a recent United States national research (2). Pharmacological treatment is part of the comprehensive multimodal evidence-based strategy to achieve adequate control of core ADHD symptoms and comorbid conditions, minimizing functional impairments and improving quality of life (3). Drug strategies for the treatment of ADHD are categorized into stimulants and non-stimulants. Although short-term efficacy of psychostimulants is well-established, the effectiveness of these drugs in the educational, vocational and social domains in long-term treatment remains uncertain (4, 5). Treatment selection strategy should also take into account aspects that impact medication treatment response such as age of the patient, severity of the disorder, and comorbidities (6).

COVID-19 has been causing huge disruptions in mental healthcare services worldwide (7). A significant increase of mental health complaints in the general population since the beginning of the pandemics have lead some authors to speculate that mental illness may be the next “inevitable pandemic” (8). This hypothesis seems to be especially relevant when we take together both the psychosocial effects of the pandemic and the vulnerability of psychiatric patients, including those with ADHD, as two different risk factors (9).

Recently investigators and clinicians have sought to examine the possible impact of COVID-19 pandemic on patients with ADHD (10–13). Cortese et al. suggest that the distress caused by the pandemic and the physical distancing measures may worse some of the behavioral problems already seen in patients with ADHD (14). Moreover, some consequences of the pandemic, such as virtual schooling and remote work, as well as increased telemedicine, have posed new challenges for the diagnosis and treatment of ADHD. For instance, initializing and optimizing medication treatments for ADHD has been a theme of great concern (15). Although some findings on this issue were already published, there is still a need for an in-depth investigation on the impact of the current health crisis over patients with ADHD.

In this narrative review, we summarize existing COVID-19-related literature pertinent to ADHD, with a special focus on treatment adherence, integrating recent research findings. We also provide a discussion on the potential implications of the reviewed studies for the ADHD field and provide future research directions.

This narrative review aims to identify which are the most relevant findings regarding the new challenges in diagnosing and treating patients with ADHD during the COVID-19 pandemic. We believe that the biggest challenge is to establish how to perform the best remote monitoring of patients with ADHD, ensuring good adherence to treatment. Using telemedicine more efficiently will be a crucial point in achieving good adherence to ADHD treatment.

## Methods

In this review, we use a narrative approach (16, 17). The aim is to summarize the main findings of the studies that investigate the impact of the COVID-19 pandemic on patients with ADHD and the role of ADHD as a risk factor for COVID-19. We also give a special focus on two issues: (1) diagnosing ADHD during the pandemic and; (2) the challenges that clinicians face when treating patients with ADHD during the pandemic. The following databases were searched using the terms ‘ADHD and COVID-19’: PubMed, PsycINFO, EMBASE, Google Scholar and medRxiv. We selected and included all English language papers and preprints resulting in a total of 546 articles. We excluded the repetitions and have selected only the ones that reported on medication/pharmacological treatment among the covered topics on the implications of COVID-19 in ADHD management. The final search was done on Dec-23 2021. We have included case reports, case–control studies, reviews, commentaries, viewpoints, perspectives, guidelines and letters to the Editor. We also searched conference abstracts for events held in 2020 and 2021, considering the rapidly evolving scope of this review.

## Results

A total of fifteen studies were identified and included in this narrative review. There were no randomized controlled trials on this specific topic. Most of the included studies were viewpoint/expert opinion, case–control studies and treatment guidelines. Table 1 provides a summary of each study included in this review. We summarized the findings of these studies under the main headings described below.

**TABLE 1:** Information on the included studies.

	Author(s)	Country	Journal	Study/Article type	Summary	Key findings
1	Singh et al. (18)	India	Psychiatry Research	Expert opinion/review	The authors discuss the potential negative impact of the COVID-19 pandemic on mental health of children and adolescents.	There is a need to ameliorate children and adolescents’ access to mental health support services geared toward providing measures for developing healthy coping mechanisms during the current crisis.
2	Surman et al. (19)	United States	Abstract APSARD 2021	Pilot	This study explored the sensitivity of electronic patient peported outcome measures to medication effects in adults with ADHD (*n* = 91) during the pandemic.	Observation of a likely correlation between patient sensitivity and mobile ADHD symptoms while changes in treatment status were monitored.
3	Becker and Gregory (20)	United States	J Child Psychol Psychiatry	Editorial perspective	The authors discuss ways in which the pandemic may impact sleep and associated psychopathology for child and adolescents.	Youth with preexisting psychopathologies and neurodevelopmental conditions (including attention-deficit/hyperactivity disorder and autism spectrum disorder) could be especially vulnerable to disturbed sleep during this period of change and uncertainty.
4	McGrath (21)	Ireland	I J Psychol Med	Expert opinion/viewpoint	The author discusses the impact of the COVID-19 pandemic on the provision of mental health services for young people with ADHD, with a focus on a specialist ADHD service in Dublin.	Current guidelines and alternative ways of ensuring adequate service provision are discussed. Factors that should be considered when developing a telepsychiatry service for children and adolescents with ADHD are highlighted.
5	Becker et al. (10)	United States	J Adolesc Health	Survey	A survey study of remote learning practices and difficulties at the beginning of the COVID-19 pandemic in adolescents with and without ADHD (*n* = 238).	Adolescents with ADHD had fewer routines and more remote learning difficulties than adolescents without ADHD. Parents of adolescents with ADHD had less confidence in managing remote learning.
6	Non-weiler et al. (11)	United Kingdom	Children	Survey	A parent-reported study using the Strengths and Difficulties Questionnaire conducted 2nd April–2nd June 2020 (*n* = 453 children aged 4–15 years).	All groups had worse emotional symptoms than pre-COVID-19 groups, and those with attention-deficit/hyperactivity disorder showed inflated conduct problems.
7	Sibley et al. (12)	United States	J Psychiatr Res	Survey	A survey study of self and parent ratings about current and pre-pandemic top problem severity and benefits of the pandemic (*n* = 134).	There was no evidence that pandemic-related changes mitigated ADHD severity.
8	Sciberras et al. (13)	Australia	J Atten Disord	Survey	A survey study with parents (*n* = 213) of children (5–17 years) with ADHD conducted in May 2020 about physical and mental health, as well as media use during the pandemic.	COVID-19 restrictions were associated with both negative and positive impacts among children with ADHD.
9	Wyler et al. (22)	Switzerland	BMC Psychiatry	Mixed-method	This study combines a quantitative questionnaire data and qualitative data from interviews to explore ADHD patients’ and therapists’ experience of a specific therapy session during the COVID-19 pandemic in one of the following three treatment modalities: face to face, videoconferencing, and telephone.	Both settings, on-site with the therapist wearing a face mask and telepsychiatry, seem to be valid options to continue treatment of adults with ADHD during a situation such as the COVID-19 pandemic.
10	Cortese et al. (14)	International	Lancet Child Adolesc Health	Guidelines	A consensus statement on ADHD management during the COVID-19 pandemic published by the European ADHD Guidelines Group.	The risks and benefits of initiating or maintaining medication under the COVID-19 restrictions implemented in some countries should be carefully considered. If the use of medication is deemed desirable, strategies for remote monitoring should be implemented.
11	Cortese et al. (15)	International	Lancet Child Adolesc Health	Guidelines	An addendum on the previous guideline about starting ADHD medications during the pandemic.	Provides additional guidance for patients who did not have a baseline, face-to-face cardiovascular assessment before the crisis began.
12	Merzon et al. (23)	Israel	J Atten Disord	Observational	The authors examined whether ADHD constitutes a risk factor for COVID-19 infection and the role of pharmacotherapy as a protective factor using patient registered data (*n* = 14,022).	The risk for COVID-19-Positive was higher in untreated-ADHD subjects compared to non-ADHD subjects, while no higher risk was detected in treated ones.
13	Breaux (24)	United States	J Child Psychol Psychiatry	Observational	The authors assessed ADHD and other mental health symptoms (*n* = 238) shortly before the COVID-19 pandemic and in spring and summer 2020.	Adolescents with ADHD were more likely than adolescents without ADHD to experience an increase in inattentive, hyperactive/impulsive, and oppositional/defiant symptoms.
14	Altena et al. (25)	France	J Sleep Res	Review	Authors reviewed the literature on the stress-sleep link and confinement, as well as effective insomnia treatment.	Managing sleep problems as best as possible during home confinement can limit stress and possibly prevent disruptions of social relationships.
15	Breaux et al. (26)	United States	The ADHD Report	Editorial report	A report on the effects of COVID 19 pandemic on ADHD care and the use of telehealth.	Evidence suggesting the increased and far-reaching risk for individuals with ADHD during the COVID-19 pandemic.

### The Impact of COVID-19 Pandemic on Mental Health, Sleep and Well-Being Outcomes in Patients With Attention Deficit Hyperactivity Disorder

The mental health outcome of the COVID-19 pandemic is increasingly recognized as a relevant, worldwide public health concern (27). In individuals with pre-existing neurodevelopmental disorders, such as ADHD, it has been hypothesized that the distress caused by the pandemic and physical distancing measures may lead to worsening of behavioral problems (14). In the current scenario, new priorities and challenges for diagnosing and treating individuals with ADHD have emerged.

Sibley et al. used a survey to assess the top problems reported by adolescents and young adults with ADHD during the COVID-19 pandemic (12). The most common top problems rated as more severe during the pandemic than in prior months were difficulties engaging in online learning, boredom and social isolation. Becker et al. (10) also conducted a survey study to examine the nature and impact of remote learning during the COVID-19 pandemic in adolescents with ADHD. The authors reported that fewer adolescent routines, higher negative affect, and more difficulty concentrating because of COVID-19 were associated with greater remote learning difficulties.

Non-weiler et al. investigated the prevalence of emotional and behavioral problems assessed by parent reports among children and young people with ADHD between April and 2nd June 2020 (11). They found that ADHD children had worse emotional symptoms and inflated conduct problems than comparable cohorts pre-COVID-19.

It has also been postulated that quality of sleep may be profoundly impacted by COVID-19, and that children and adolescents with ADHD may be particularly vulnerable to disturbed sleep during the pandemic (20, 25). Breaux et al. examined changes in adolescent sleep before and during the COVID-19 pandemic using surveys with parents and adolescents with ADHD (24). Analysis of the survey answers showed that adolescents with ADHD did not experience an increase in night sleep duration in school days and were less likely to obtain recommended sleep duration during COVID-19 compared to non-ADHD controls. The majority of ADHD participants (83%) were already using medication to treat ADHD before the beginning of the pandemic. In his perspective piece about the impact of the pandemic on a tertiary-level specialist ADHD service in Dublin, McGrath describes many sleep problems reported by families (21). The author states: “…Sleep patterns had deteriorated for the majority of families, with sleep onset times pushed forward by approximately 3 h.”

Results from a survey study showed that restrictions imposed by the pandemic may also impact children and adolescent’s well-being. The authors found children and adolescents had less time spent on physical exercising, less time to engage on outdoor activities and less enjoyment in those activities while time spent on television, social media, gaming, sad/depressed mood, and loneliness were increased compared to pre-pandemic era (13).

### the Impact of the COVID-19 Pandemic on Diagnosing and Treating Attention Deficit Hyperactivity Disorder: Challenges on Treatment Adherence

The implementation of a medication protocol in ADHD with periodic assessment of symptoms, real-life functional benefits and adverse effects is imperative for adjusting pharmacological treatment to optimize outcomes (6). During the COVID-19 pandemic, the EAGG released best practice recommendations suggesting individuals with ADHD should continue with medication as usual (14). However, it remains unclear whether government restrictions in different countries have prevented these recommendations from being followed by telepsychiatry.

An addendum of the EAGG recommendations was released with additional advice on starting ADHD medications during the 2019 pandemic for individuals with ADHD who did not have a pre-pandemic baseline in-person cardiovascular assessment (15). They recommended that, given the circumstances imposed by the pandemic, cardiac auscultation should not be mandatory in individuals with no risk factors for cardiac disease. The group suggested that baseline monitoring (i.e., blood pressure and heart rate measured in three separate occasions) before medication initiation can be done by a lay person supervised through remote assistance. Evidence for the effectiveness of telepsychiatry in the treatment of ADHD comes from a recent systematic review with a limited number of studies (*n* = 11) suggesting this modality as a viable method to provide pharmacologic treatment for children with ADHD (28). Thus, there is a strong need for studies examining the effects of remote ADHD-medication prescriptions during the COVID-19 pandemic on treatment outcomes.

Changes in medication treatment patterns during the pandemic were investigated by Sciberras et al. in a survey study with 213 parents of children with ADHD in Australia (13). Data were gathered during a 4-week period in May 2020 when social distance measures (i.e., requiring citizens to stay at home except for essential reasons) were in place. Approximately two-thirds of the parents reported that their child was taking medication, mostly stimulants, to assist with learning, behavioral, emotional or sleep difficulties. When the parents of those children taking medication were asked specific questions about medication use in the last month, 17% reported a dosage change (majority had increased dosage), and 16% reported that their child had stopped taking a medication. Most of the stopped medications were ADHD medications (*n* = 24). Among the reasons informed by parents for stopping an ADHD medication were taking a break during school holidays, not requiring medication due to school closure/remote learning and stopping one ADHD medication to start another. Only 11% of parents reported difficulties in purchasing ADHD medication. Among the reasons, the two most common were: (1) medication not available on the stock and (2) difficulties on getting the prescriptions.

In his perspective article, McGrath discusses the challenges to optimizing ADHD medication for children and adolescents in face of school closures (21). He points out the difficulties to determine the effectiveness of medication without teacher feedback during medication titration. The author emphasizes the role of parental report in this process, since many parents have been working from home and then have a unique opportunity to comment on the effectiveness and impact of medication on their child’s academic and social functioning.

In another survey study from Switzerland, Wyler et al. explored the perceptions of therapists and adults with ADHD about three different modalities of therapy sessions (i.e., face-to-face with the therapist wearing a face mask, *via* telephone, or videoconferencing) during the COVID-19 pandemic (22). Qualitative analysis of responses showed that patients felt that a telephone session worked well to discuss medication. Therapists reported that the limitations of a videoconferencing session were less important for sessions that focused on medication rather than psychotherapeutic work.

Despite the challenges posed by the pandemic on monitoring ADHD treatment, the current scenario also may offer new opportunities that could lead to the development of “targeted” and individualized pharmacological interventions. This issue has been considered by a recent pilot work with 90 adult patients taking stimulant therapy for ADHD (19). The study collected demographic information, medication use history and patterns, and symptoms associated with ADHD using mobile phone surveys. The researchers adopted an individualized approach to send patients medication-sensitive items of the Weiss Functional Impairment Rating Scale (WFIRS) and the Adult ADHD Self Report Scale v1.1 (ASRS) during selected time periods in the morning and evening. From these data, investigators noted sensitivity thresholds for symptom reporting by patients, as well as within-day and between-day differences in response patterns. Researchers then inferred “on” and “off” medication status based on mobile monitoring of ADHD symptoms and functional impact.

Moreover, there is now an opportunity to exploit the wider use of digital medicine innovations on monitoring patients that are using medication. This perspective is discussed by McGrath, who points to the widespread inclusion of heart rate monitors in smartphones and fitness watches as well as the possibility of inferring it from contact photoplethysmography (PPG) using cell phone cameras (21). The author argues that home-monitoring of heart rate is currently a feasible option, since most families have access to a smartphone.

### Attention Deficit Hyperactivity Disorder as a Risk Factor for COVID-19 and the Role of Attention Deficit Hyperactivity Disorder’s Pharmacological Treatment

It has been postulated that ADHD-related difficulties are associated with a high risk of having COVID-19 (18). To test this hypothesis, Merzon et al. (23) analyzed data from 14,022 people in Israel registered to a comprehensive database between February 1st and April 30, 2020, who underwent at least one COVID-19 test (23). The researchers reported an infection rate of 10.1% in the total sample. Analyzing the sample of individuals with a positive test result, the authors reported that these individuals were younger and had higher rates of ADHD diagnosis when compared with the sample of individuals that tested negative for COVID-19. The hypothesis of ADHD also increases the risk of severe COVID-19 infection was studied as well. The authors found that ADHD was associated with poorer outcomes in COVID-19 infection (29). In another study, people with ADHD were more vulnerable to the challenges created by the COVID-19 pandemic (30). In a report about the impact of COVID-19 on ADHD patients, the authors highlights the heterogeneity of risk among individuals with ADHD (11).

To further investigate the effect of ADHD stimulant pharmacotherapy on preventing COVID-19 infection in patients with ADHD, the authors carried out subsequent comparisons between patients treated for ADHD, patients not treated for ADHD and a control group of individuals with no ADHD diagnosis (23). Results showed that the risk for COVID-19 was higher in ADHD patients not treated. They concluded that stimulant pharmacotherapy may reduce the risk of COVID-19 infection in patients with ADHD.

## Discussion

In this paper, we review the current state of knowledge on the potential impact on the COVID-19 pandemic for the clinical management of ADHD. The key findings in this review are: (1) Patients with ADHD had a higher prevalence of psychiatric symptoms as a consequence of the COVID-19 pandemic. There was also a greater negative impact on learning, social life, and quality of life; (2) The risk of discontinuing the treatment of ADHD, with negative consequences for patients and families, has brought a warning to medical societies. This concern led to the creation of guidelines and strategies to maintain the correct monitoring of these patients at a distance; (3) Research findings showed a higher frequency of ADHD diagnoses in samples from COVID-19 patients. Correct treatment of ADHD was associated with a lower risk of SARS Cov 2 infection. The main findings summarized above describe the many negative consequences associated with stopping ADHD treatment. They also point to the difficulties of evaluating and diagnosing new symptoms or comorbidities. Finally, the findings suggest the need to maintain remote monitoring, especially using telemedicine. Several studies have been published to evaluate the effectiveness of telemedicine. Positive results regarding its effectiveness, but also its limitations, have been investigated for decades (31, 32). The COVID-19 pandemic has increased the importance of telemedicine in medical practice. Telemedicine is expected to deliver timely care while minimizing exposure to protect medical practitioners and patients (33). So the use of telemedicine and virtual software offers promising potential in the fight against COVID-19 (34). A large amount of research in this area has clarified old questions, increased our knowledge and brought new challenges (35). In a recent article on the subject, the international consortium REPROGRAM provided a complete guide for the implementation of telemedicine during the COVID-19 pandemic and the relevance of its maintenance after this pandemic period (36).

The use of telemedicine for the diagnostic evaluation of patients with a clinical picture suggestive of ADHD has already been investigated. In a review on the subject, carried out before the COVID-19 pandemic, the authors indicated the effectiveness of telemedicine in the evaluation of possible new cases of ADHD. These findings are especially important for the evaluation of patients in remote areas where specialists are not present (28). However, the effectiveness of telemedicine for the follow-up of patients already diagnosed and on medication for ADHD has not been evaluated. The beginning of the COVID-19 pandemic brought the need to develop new research to evaluate the effectiveness in the follow-up of patients already diagnosed with ADHD.

Monitoring ADHD patients during the COVID-19 pandemic represents a challenge for specialists and general practitioners. The findings indicate that the discontinuation of psychotherapy and pharmacological treatment is very harmful to patients. Guidance on the best way to monitor patients remotely has been provided by several medical associations. However, we still do not have enough specific research on the effectiveness of remote monitoring of patients with ADHD. At this time, further research to investigate the role of telemedicine in the follow-up of patients with ADHD (and not just for initial diagnosis) is needed.

## Conclusion

The COVID-19 pandemic has brought new challenges to the treatment of patients with ADHD. Initial findings showed that ADHD patients during the COVID-19 pandemic are more vulnerable to a variety of negative outcomes. These patients present greater difficulties in studies and school performance. The various findings presented in this review point to the need to adequately maintain the treatment of individuals with ADHD. Strategies for treatment adherence are even more necessary. The use of telemedicine, in particular, proved to be a very relevant measure.

Our review has limitations that need to be highlighted. The choice of narrative review instead of systematic review is the main limitation. We recognize that a systematic review would be the best choice. However, carrying out a systematic review in our study center in such a short time proved to be unfeasible. Another limitation that should be pointed out is inherent to the selection of reviewed articles. In order to focus mainly on articles on monitoring patients with ADHD in the context of the COVID-19 pandemic, we excluded articles and reviews that included samples from patients diagnosed with other mental disorders. Finally, another important limitation was the inclusion of only articles published in English during the years 2020 and 2021.

Future research should focus on conducting prospective follow-up studies of patients with ADHD and their families, using telemedicine strategies. These new studies may provide more detailed data on both short-term and long-term adherence to ADHD treatment. They will also be able to point out which changes and adaptations will be necessary for better monitoring of patients with ADHD.

## Author Contributions

DS defined the scope of publication interpreted data, wrote the manuscript, screened the studies, analyzed the data, provided a critical review of the manuscript, revised critically the work providing substantial input, and gave final approval of the version to be published.

## Conflict of Interest

DS has served on the speaker’s bureau and/or acted as consultant for Shire/Takeda; he has received travel awards to participate in scientific meetings from this company.

## Publisher’s Note

All claims expressed in this article are solely those of the authors and do not necessarily represent those of their affiliated organizations, or those of the publisher, the editors and the reviewers. Any product that may be evaluated in this article, or claim that may be made by its manufacturer, is not guaranteed or endorsed by the publisher.
